# The impact of the COVID-19 crisis on meeting needs for family planning: a global scenario by contraceptive methods used

**DOI:** 10.12688/gatesopenres.13148.2

**Published:** 2020-11-04

**Authors:** Aisha Dasgupta, Vladimíra Kantorová, Philipp Ueffing

**Affiliations:** 1 United Nations Population Division, Department of Economic and Social Affairs, United Nations, 2 United Nations Plaza, New York, NY, 10017, USA

**Keywords:** COVID-19, family planning, demand for family planning satisfied by modern methods, Sustainable Development Goals, contraceptive methods, scenario, global

## Abstract

The COVID-19 crisis could leave significant numbers of women and couples without access to essential sexual and reproductive health care. This research note analyses differences in contraceptive method mix across Sustainable Development Goal regions and applies assumed method-specific declines in use (from 0 per cent to 20 per cent) to produce an illustrative scenario of the potential impact of COVID-19 on contraceptive use and on the proportion of the need for family planning satisfied by modern methods. Globally, it had been estimated that 77 per cent of women of reproductive age (15-49 years) would have their need for family planning satisfied with modern contraceptive methods in 2020. However, taking into account the potential impact of COVID-19 on method-specific use, this could fall to 71 per cent, resulting in around 60 million fewer users of modern contraception worldwide in 2020. Overall declines in contraceptive use will depend on the methods used by women and their partners and on the types of disruptions experienced. The analysis concludes with the recommendation that countries should include family planning and reproductive health services in the package of essential services and develop strategies to ensure that women and couples are able to exercise their reproductive rights during the COVID-19 crisis.

## Disclaimer

The views expressed herein are entirely those of the authors and do not necessarily reflect the views of the United Nations.

## Introduction

Declared a global pandemic in March 2020, COVID-19 has come to affect the lives of billions of people around the world. It is the largest global public health emergency since the Spanish flu pandemic of 1918–1919 and has put many countries’ health-care systems under severe stress. Most governments have responded by introducing far-reaching policies, including behavioural changes aimed at limiting transmission and saving human lives. This has impacted a multitude of sectors, including sexual and reproductive health care, for which an essential component is the provision of safe, effective, affordable and acceptable methods of contraception. The COVID-19 crisis could leave significant numbers of women and couples without access to essential sexual and reproductive health care.

COVID-19 is impacting women’s ability to use contraception in a number of ways: disruptions to the supply chain are limiting the production, distribution and availability of contraceptive commodities, resulting in stock-outs ( Purdy, 2020); some health-care facilities are reducing services ( IPPF, 2020; MSI, 2020a); health-care providers are redirected from providing family planning services towards responding to COVID-19 ( Santoshini, 2020); and many women are unable to visit health-care facilities due to lockdowns or fear of exposure to COVID-19 ( UNFPA, 2020). When women’s and couple’s needs for family planning are not met, the number of unintended pregnancies is certain to rise, with life-long impacts on women and their families.

Riley *et al.* (2020) produced a scenario of 10 per cent decline in the use of short- and long-acting reversible contraception in low- and middle-income countries due to COVID-19, which resulted in an additional 49 million women with unmet need for modern contraception and an additional 15 million unintended pregnancies over the course of the year in low and middle-income countries. United Nations Population Fund ( UNFPA, 2020) and Avenir Health modelled a range of scenarios of unmitigated impact in 114 countries covering 93 per cent of users in low- and middle-income countries and projected 47 million women to be unable to use modern contraceptives due to the COVID-19 disruptions continuing for six months. It is possible that these scenarios provide conservative estimates of the global impact, since service providers have suggested even larger disruptions to services in 2020 ( MSI, 2020b). These scenarios were produced for a selection of countries and limited to estimates of contraceptive use changes and their impact on a range of outcomes, such as unintended pregnancies, unsafe abortions and maternal deaths.

**Figure 1.**
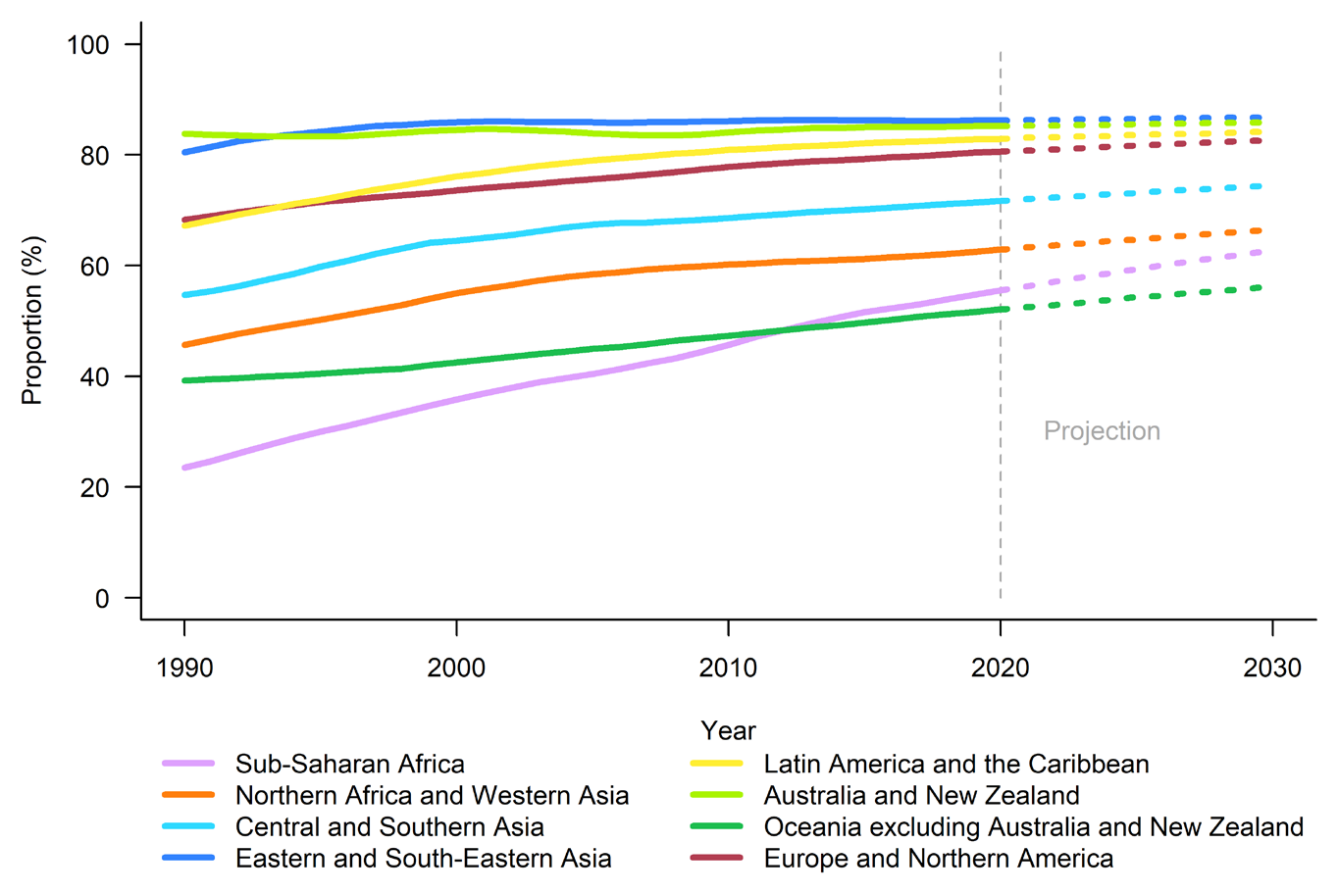
Trends in the proportion of women of reproductive age (15–49 years) who have their need for family planning satisfied with modern methods. *Source*: United Nations, Department of Economic and Social Affairs, Population Division (2020). *Estimates and Projections of Family Planning Indicators 2020.* This figure is reproduced here under a Creative Commons Attribution 3.0 IGO license.

This research note presents a scenario of the impact of COVID-19 on the Sustainable Development Goals (SDG) indicator 3.7.1., the proportion of women who have their need for family planning satisfied by modern methods. The indicator is monitored annually by the United Nations Population Division. Globally, it was estimated that the proportion of women of reproductive age (15–49 years) who had their need for family planning satisfied with modern contraceptive methods increased slightly, from 74 per cent in 2000 to 77 per cent in 2020 ( United Nations, 2020a) ( Figure 1). It is projected to reach 78 per cent in 2030, with a 95 per cent uncertainty interval of 74 per cent to 81 per cent. Just half of the need is satisfied with modern methods in sub-Saharan Africa today.

The impact of the COVID-19 pandemic on meeting the demand for family planning will be influenced by many factors, one of them being the types of contraceptive methods used by women in each country. Individual contraceptive methods differ in terms of the need for contact with health-care providers, the periodicity of renewal, the susceptibility to stock-outs and global supply chains disruptions, and their effectiveness in preventing unintended pregnancies.

**Figure 2.**
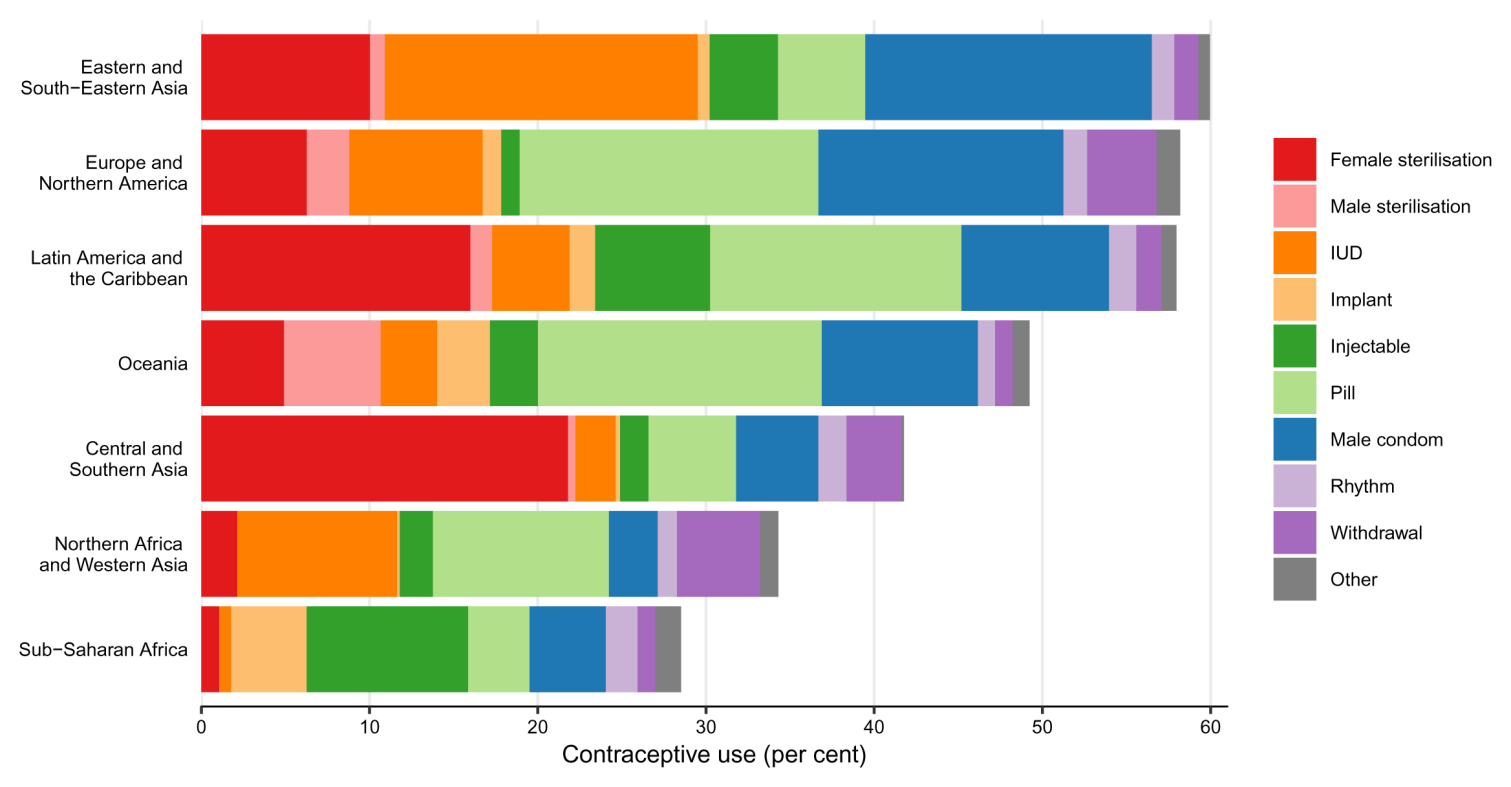
Contraceptive use by method among women of reproductive age (15–49 years), by region, in 2019. *Source*: United Nations, Department of Economic and Social Affairs, Population Division (2019). *Contraceptive use by method 2019.* *Note:* In this figure, Oceania includes Australia and New Zealand. *Abbreviations:* IUD, intrauterine device. This figure is reproduced here under a Creative Commons Attribution 3.0 IGO license.

Estimates of contraceptive use by individual methods are available at the national, regional and global levels ( United Nations, 2019). The prevalence of use of different contraceptive methods varies widely by region ( Figure 2). For example, in Central and Southern Asia the most common method is female sterilisation (22 per cent of women of reproductive age rely on this method), while injectables are the dominant method in sub-Saharan Africa, with a prevalence of 9 per cent among women of reproductive age.

## Methods

This research note analyses the differences in contraceptive method mix across regions and applies method-specific declines in use to produce an illustrative scenario of potential impact of COVID-19 on contraceptive use and the proportion of need for family planning satisfied by modern methods. The scenario is implemented in Microsoft Excel and is provided as *Underlying data* ( Dasgupta *et al.*, 2020).

Contraceptive prevalence is the percentage of women who report themselves or their partners as currently using at least one contraceptive method. Unmet need for family planning is the percentage of women who want to stop or delay childbearing for at least two years but are not using any contraceptive method. Demand for family planning satisfied by modern methods (SDG indicator 3.7.1.) is modern contraceptive prevalence divided by total demand, which is the sum of contraceptive prevalence and unmet need.

The methods for national, regional and global estimates and projections of family planning indicators among women of reproductive age (15–49 years) used in this research note are described in Kantorová *et al.*, 2020. The estimates and projections are available from 1990 to 2030 for all women of reproductive age (15–49) for 186 countries or areas with a total population of 90,000 people or more and with at least one observation of contraceptive prevalence, as well as for aggregate geographic regions ( United Nations, 2020a). They are weighted by population and take into account changes in marital status. The survey data underlying the model-based estimates and projections are publicly available as a comprehensive data set of 1,317 survey-based observations for 196 countries or areas for the period 1950 to 2019 ( United Nations, 2020b).

We applied the method-mix ( United Nations 2019) to the contraceptive prevalence in 2020 ( United Nations, 2020a) to produce estimates of method-specific prevalence for 2020. These were then reduced by method-specific declines ( Table 1). The resulting modern contraceptive prevalence was divided by total demand ( United Nations, 2020a), in order to produce a scenario of demand for family planning satisfied by modern methods.

**Table 1.** Scenario assuming declines in use by method, with justification.

Method	Assumed percentage decline in use	Justification
Female sterilization	2%	Some existing female sterilization users age-out and are not replaced by adopters of sterilization. We applied the 20% decline to this fraction, estimated as 1/10, because the CYP for female sterilization is 10.
Male sterilization	2%	Some wives/partners of existing male sterilization users age-out and are not replaced by adopters of sterilization. We applied the 20% decline to this fraction, estimated as 1/10, because the CYP for male sterilization is 10.
IUD	4.3%	Some existing IUD users age-out or discontinue their use because the commodity expires. We applied the 20% decline to this fraction, estimated as 1/4.6, because the CYP for Copper T IUD is 4.6. The assumed decline is an overestimate, since existing users who require a resupply are likely to still be somewhat protected by their expired IUD.
Implant	5.3%	Some existing implant users age-out or discontinue their use because the commodity expires. We applied the 20% decline to this fraction, estimated as 1/3.8, because the CYP for *Jadelle* implant is 3.8. The assumed decline is an overestimate, since existing users who require a resupply are likely to still be somewhat protected by their expired implant.
Oral contraceptive pills	10%	Pills can be accessed from a variety of sources (e.g. pharmacies) with limited interaction with health care system. The 10% decline is consistent with UNFPA (2020) medium public sector scenario.
Condoms	10%	Condoms can be accessed from a variety of sources and distribution channels. Access does not require interaction with health care system. The 10% decline is consistent with UNFPA (2020) medium public sector scenario.
Injectables	20%	With the exception of self-injectables (e.g. Sayana press), the majority of injectable users require interaction with a service provider. This interaction is typically required every three months, so discontinuation is likely to be more heavily impacted by COVID-19. The 20% reduction is consistent with UNFPA (2020) medium public sector scenario.
Other modern methods (including vaginal barrier methods, emergency contraception)	10%	Consistent with UNFPA (2020) medium public sector scenario.
Lactational amenorrhea method	0%	No decline assumed since women can use this method without contraceptive commodity / service provision
Traditional methods including rhythm, withdrawal	0%	No decline assumed since women can use this method without contraceptive commodity / service provision. Any change in traditional use does not affect SDG 3.7.1. since the indicator is concerned with demand satisfied by modern methods.

CYP, couple years of protection; IUD, intrauterine device; UNFPA, United Nations Population Fund; SDG, Sustainable Development Goal.

The scenario assumes no change in sexual activity, fertility intentions, or total demand for family planning, as was also assumed by Riley *et al.* (2020), and no difference in the impact of COVID-19 disruptions on married/in-union versus unmarried women.

The assumed percentage decline in use for each method is presented in Table 1. Where possible, we followed the UNFPA (2020) assumptions of service disruption according to their public sector medium scenario. Broadly, we assume a 10 per cent decline for commodities that can be sourced from a range of distribution channels (e.g. condoms and oral contraceptive pill), and a 20 per cent decline for methods that require a service provision from a health care provider (e.g. injectables). For long-acting and permanent methods, all of which require a service from a health care provider, we use the metric couple years of protection (CYP) ( USAID, 2019) to estimate the number of users requiring a service over the course of the year to maintain the existing number of users, and apply the 20 per cent decline to that portion. No decline was assumed for methods that do not require any commodity or contact with a service provider. Because of the lack of country-specific data, we assume no differences in method-specific decline across countries and regions, despite the fact that some countries and regions may be better prepared to handle the crisis than others. We also make no assumptions about switches between modern methods of contraception (e.g. from injectables to oral contraceptive pills or condoms). This does not mean there is no method-switching. Indeed, it is very possible that some women will switch from some methods to other methods, depending on their own context and the way that COVID-19 impacts specific methods in specific countries. Rather, the declines are an indication of the overall decline in use by method, after method-switching.

Regarding the time-frame of our analysis, we assume the disruptions take place over the year 2020 and calculate the estimates for this year. While the disruptions might not be equally spaced through 2020, we assume the impact is averaged over the year.

As a sensitivity test, we also prepared a separate scenario using an assumed 10 per cent decline in short-term and long-acting reversible contraception (the scenario is included in *Underlying data*) ( Dasgupta *et al.*, 2020). This replicates the Riley *et al.* (2020) approach, but extends their analysis to the global level, and to the indicator demand for family planning satisfied by modern methods.

## Results

**Figure 3.**
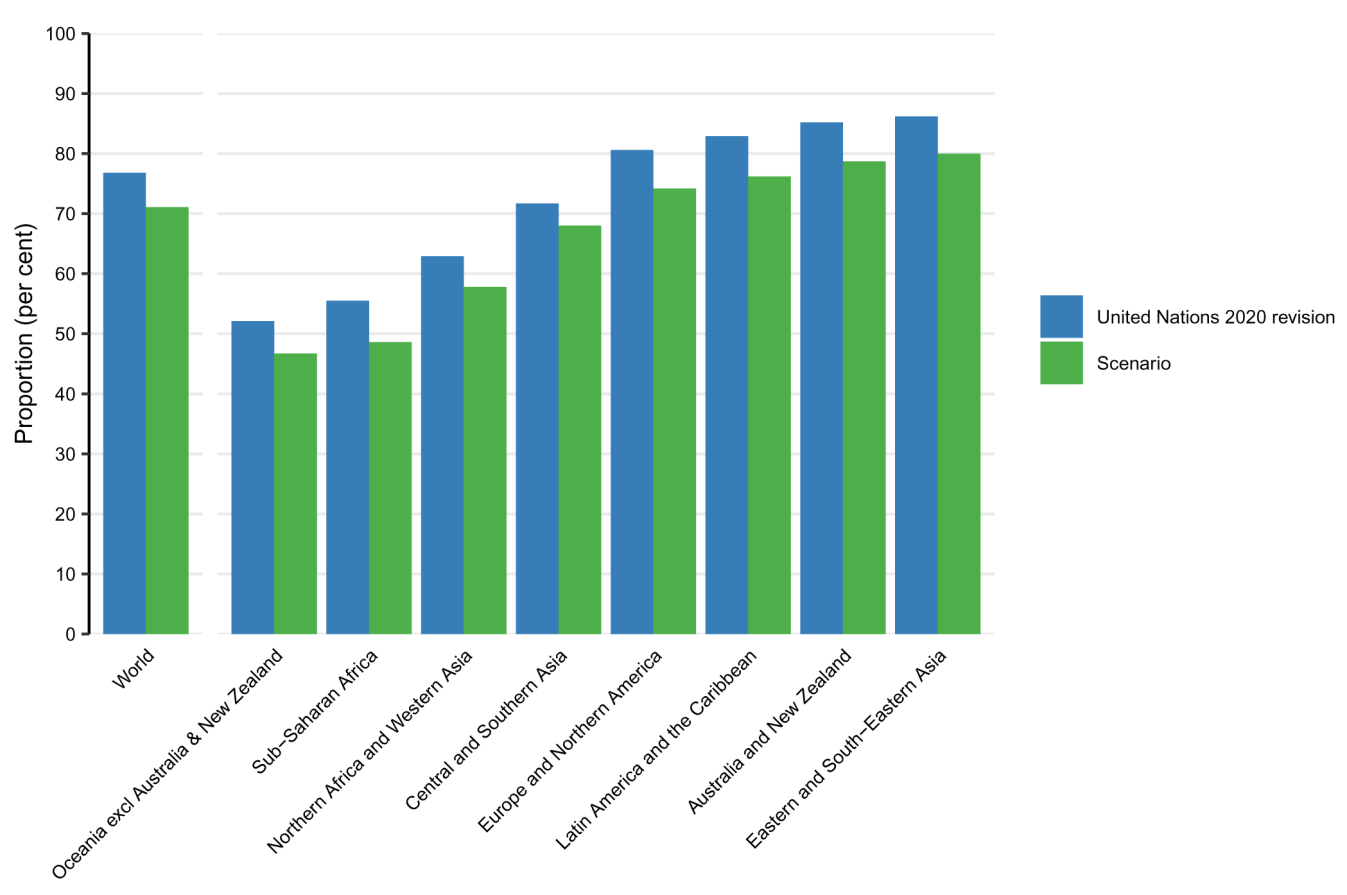
Proportion of women of reproductive age (15–49 years) who have their need for family planning satisfied with modern methods in 2020.

In the scenario of method-specific declines, the proportion of women who have their need for family planning satisfied with modern methods could fall to 71 per cent in 2020 ( Figure 3), which would be a regression to levels not seen at the global level since 1995. The largest declines would be in Latin America and the Caribbean (6.7 percentage points) and sub-Saharan Africa (6.8 percentage points), because these regions have a method-mix skewed towards short-term methods. Central and Southern Asia would experience a smaller average decline (3.7 percentage points), because this region has a high proportion of women using female sterilization, which is least affected by short-term disruptions.

Under this scenario, the impact of the pandemic could be around 60 million fewer users of modern contraception worldwide in 2020.

Under the separate scenario with an assumed 10 per cent decline in all short-term and long-acting reversible contraception in line with the approach taken by Riley *et al.*, 2020, the estimate of the need for family planning satisfied with modern methods in 2020 is 72 per cent. The difference to the method-specific decline scenario is small at the global level. 

Overall declines in contraceptive use will depend on the methods used by women and their partners, and the types of disruptions experienced (availability of commodities, health care service provision). For example, countries with a high prevalence of long-acting and permanent methods (LAPM) would likely experience little change, as many LAPM users will continue to be protected. On the other hand, countries that rely more heavily on short-term methods such as injectables, requiring repeated contact with a service provider, would likely see a decline in use.

## Conclusions

This scenario is intended to be illustrative of the potential impacts during 2020 of continued disruptions due to COVID-19 isolating the effect of differences in type of contraceptive methods used by women or their partners across regions. Although we applied simplified assumptions across all countries, not all countries will experience the same level of disruptions due to COVID-19. We have assumed that such disruptions could last for a full year, but a shorter disruption would obviously have less impact. For example, a six-month disruption would result in half the impact (the demand for family planning satisfied by modern methods in 2020 would be 74 per cent).

The COVID-19 pandemic has made the path towards achieving universal access to sexual and reproductive health-care services by 2030, including family planning, more uncertain. Once the disruptions due to COVID-19 are resolved, it is possible that contraceptive use – and therefore the SDG 3.7.1. indicator – could return to the pre-disruption levels relatively quickly. For short-term method users, assuming that methods require frequent resupply, the health system could in principle recover to pre-COVID-19 levels within a short period of time, once service activities have fully resumed. However, for long-acting methods, there could be a longer period to catch up on services that were not provided during COVID-19 disruptions.

Most crucially, for women – and their partners and families – who experienced an unintended pregnancy resulting from the lack of access to contraception during COVID-19 disruptions, the impacts are long-lasting. To understand the impacts of COVID-19 disruptions on contraceptive services and use, countries and family planning service providers need to continue data collection through health management information systems, focusing on data quality and completeness during the crisis. This is especially needed because major survey programmes paused the data collection field work. By the time surveys have resumed, some of the gaps in contraceptive use may have recovered, as shown by research on contraceptive use during and after the West African Ebola crisis ( Bietsch *et al.*, 2020), and so the decline in use during the crisis might not be observed in future surveys. The data from health management information systems and information about supply chains will also help to inform projections for year 2030.

While we do not yet know how fertility preferences will change in response to the COVID-19 crisis, it is likely that as a result of the economic downturn and increasing uncertainties, some women and couples who were planning a pregnancy may decide to postpone childbearing to a later period as was already documented for the United States ( Lindberg *et al.*, 2020). These changes in childbearing preferences increase the need for family planning methods, and therefore our scenario could underestimate the potential impact of COVID-19. However, Aassve *et al.* (2020) hypothesized that — depending on the development level of the country — the changes in fertility preferences might not be present in all countries. Additionally, the frequency of sexual activity during the COVID-19 pandemic could have been impacted by health and behavioural changes. We did not explicitly include the changes in fertility preferences and sexual activity in the scenario, since our main purpose was to illustrate the impact of method-specific access disruptions.

Countries should include family planning and reproductive health services in the package of essential services and develop strategies to ensure that women and couples are able to exercise their reproductive rights during the COVID-19 crisis. WHO recommends practical actions that countries can take at national, subregional and local levels to reorganize and safely maintain access to high-quality, essential health services in the pandemic context ( WHO, 2020; on page 29), such as relaxation of requirements for a prescription for oral or self-injectable contraception and emergency contraception and provision of multimonth supplies with clear information about the method and how to access referral care for adverse reactions, and enabling pharmacies and drugstores to increase the range of contraceptive options they can provide. For the transition towards restoration of the services, it recommends to plan for clients whose choice are longer-term methods (such as IUDs, implants) or permanent methods, if these services were disrupted. The experience gained during the present pandemic should be used to develop preparedness and contingency plans for any future disruptions.

## Data availability

### Source data

Contraceptive prevalence data was retrieved from the United Nations, Department of Economic and Social Affairs, Population Division *Estimates and Projections of Family Planning Indicators 2020* ( https://www.un.org/development/desa/pd/node/3288) and United Nations, Department of Economic and Social Affairs, Population Division *Contraceptive Use by Method 2019: Data Booklet* ( https://www.un.org/development/desa/pd/sites/www.un.org.development.desa.pd/files/files/documents/2020/Jan/un_2019_contraceptiveusebymethod_databooklet.pdf).

Source data are available under the terms of the Creative Commons Attribution 3.0 IGO license (CC BY 3.0 IGO).

### Underlying data

Harvard Dataverse: Supplementary material to "The impact of the COVID-19 crisis on meeting needs for family planning: A global scenario by contraceptive methods used". https://doi.org/10.7910/DVN/C6V7PN ( Dasgupta *et al.*, 2020).

Data are available under the terms of the Creative Commons Zero "No rights reserved" data waiver (CC0 1.0 Public domain dedication).
