# Supplementary material for: The impact of the COVID-19 crisis on meeting needs for family planning: a global scenario by contraceptive methods used
Source: Gates Open Res. 2020 Nov 4;4:102. Originally published 2020 Jul 9. [Version 2] doi: 10.12688/gatesopenres.13148.2 (PMC7720714; doi:10.12688/gatesopenres.13148.2)
Supplement: Peer review history

# Peer review: Reviewer response for version 2

**DOI:** 10.21956/gatesopenres.14388.r29937

**Published:** 2020-12-03

**Authors:** Mengjia Liang

**Affiliations:** 1 Technical Division, United Nations Population Fund (UNFPA), New York City, NY, USA

I went through the authors' detailed responses to my review and believe that they sufficiently addressed my comments. Therefore, I'm happy to change the status to 'Approved'.

Is the work clearly and accurately presented and does it cite the current literature?

Yes

If applicable, is the statistical analysis and its interpretation appropriate?

Not applicable

Are all the source data underlying the results available to ensure full reproducibility?

Yes

Is the study design appropriate and is the work technically sound?

Yes

Are the conclusions drawn adequately supported by the results?

Yes

Are sufficient details of methods and analysis provided to allow replication by others?

Yes

Reviewer Expertise:

NA

I confirm that I have read this submission and believe that I have an appropriate level of expertise to confirm that it is of an acceptable scientific standard.

# Peer review: Reviewer response for version 1

**DOI:** 10.21956/gatesopenres.14340.r29088

**Published:** 2020-08-06

**Authors:** Elizabeth A. Sully, Taylor Riley

**Affiliations:** 1 Guttmacher Institute, New York City, NY, USA

This research note adds to the growing model-based evidence on the disastrous indirect impacts that COVID-19 may have on essential sexual and reproductive health services. The researchers extend on prior work by producing global estimates for all countries (as compared to only low- and middle-income countries), focus particularly on the impact on the Sustainable Development Goal 3.7.1 (demand for family planning satisfied with modern methods), and expand on earlier scenarios of method-specific service reductions to account for disruptions in new users and renewal of long-acting and permanent methods. These are important contributions in drawing attention to the impact of COVID-19 on SRHR-related development goals. 

 We had a few suggestions for how the authors could (1) further strengthen the underlying analysis, (2) frame the results to better center the impacts on individuals, and (3) make the underlying data available at the country-level.  

- (1) While the prior modeling work on the impacts of COVID-19 on family planning were done in the early stages of the pandemic, this paper is coming out at a time when we are starting to know more about how services are being impacted across countries, and also on how well some of the original assumptions made by Riley et al. (2020) and UNFPA/Avenir (2020) hold up. As such, it would be great to see this paper tackle some of those underlying assumptions, either in the analysis itself, or through incorporating relevant literature.  
   
  - Changes in demand: While prior work assumed no changes in fertility preferences, the authors hypothesize in the conclusions that couples may postpone childbearing. Since the SDG indicator of primary interest in this analysis is demand satisfied, it seems worth exploring how shifts in the denominator might also impact SDG 3.7.1. Could you build another scenario looking at how demand will likely shift? To do this you could draw from recent publications on an increased desire to delay or postpone childbearing in the US ( https://www.guttmacher.org/report/early-impacts-covid-19-pandemic-findings-2020-guttmacher-survey-reproductive-health#), or other work laying out a theoretical model for how we might expect fertility to vary depending on regional income level (see Aassve *et al.*, 2020 ^1^). 
  - Method switching: If the disruptions in services are assumed to be greater for certain methods such as injectables, is it reasonable to assume that the full 20% of users would stop using any modern method entirely? It would be interesting to account for some degree of method switching, or show how sensitive the overall reductions in indicator 3.7.1. are to whether or not method-switching occurs. It would also be worth noting in the conclusions that in the face of disruptions to supply-based methods, women may at least be adopting other non-supply methods that afford them some protection from unintended pregnancy over non-use. 

 

- (2) The authors note in the conclusions that prior research from the Ebola outbreak in West Africa showed a recovery back to prior levels of contraceptive use. This is an important point that we think is worth elaborating on more, and using in the framing of how the results are interpreted. The goal of this paper is to show the potential impact on the SDG indicator 3.7.1, and the effect of COVID-19 on our development goals. However, if we observe a similar trend to that of West Africa post-Ebola, it’s possible that we could see a complete reversal on these declines in demand satisfied, putting us back on target with our development goals. The potential for a short, but not a long term impact on this goal is important to discuss further. It also highlights the need to frame the results more on the impact on women and couples, as opposed to just the indicator. While we could see a recovery in the indicator, the millions of women unable to access contraceptive methods in the intervening time will experience very real consequences of this disruption. It would be great to see the authors stress this point more, and the importance of looking not only at the SDG indicator, but also considering the impact of even short-term disruptions in coverage on individual outcomes.  

- (3) If it is possible, we urge the authors to consider releasing not just the underlying regional data for the analysis, but also the country-level data and underlying excel workbook. This would be a really valuable tool for use by stakeholders in countries who are monitoring their progress on the SDGs during this pandemic. 

Other minor recommendations to consider: 

- Abstract: It would help to provide detail in the abstract about the scenario itself, and the range of declines assumed across methods. This is important information with which to contextualize the decline in SDG indicator 3.7.1.    
- Conclusions: The last point in the conclusion suggests improving access to LARCs because these methods are less impacted. This recommendation takes away from the importance of method choice and what people want to use. While LARCs are less impacted from disruptions, we don’t want the unintended consequence of programs/countries just focusing on LARC access at the detriment to other methods to ensure choice.

Is the work clearly and accurately presented and does it cite the current literature?

Yes

If applicable, is the statistical analysis and its interpretation appropriate?

Not applicable

Are all the source data underlying the results available to ensure full reproducibility?

Yes

Is the study design appropriate and is the work technically sound?

Yes

Are the conclusions drawn adequately supported by the results?

Yes

Are sufficient details of methods and analysis provided to allow replication by others?

Yes

Reviewer Expertise:

Sexual and reproductive health, modeling impacts and costs of family planning services, abortion measurement

We confirm that we have read this submission and believe that we have an appropriate level of expertise to confirm that it is of an acceptable scientific standard, however we have significant reservations, as outlined above.

## References

[1] : The COVID-19 pandemic and human fertility. *Science*.2020;369(6502) : 10.1126/science.abc9520 370-371 10.1126/science.abc9520 Reference source 32703862

# Response

**Published:** 2020-08-28

**Authors:** Vladimira Kantorova

**Affiliations:** United Nations, USA

Dear Elizabeth and Taylor,

We thank you for the very helpful comments on the paper.

Because some of the issues were raised by more than one of the reviews, we reply to some of the common themes first. The remaining comments are included below.

**Changing total demand for family planning:**

All reviewers commented on the possibility of changing demand, either by changes in fertility preferences or sexual activity, or both. We agree with the reviewers that the changes in demand for family planning during COVID-19 could have substantial impact on the values of the indicator of demand for family planning satisfied by modern methods. We explored this at length, but decided this is not something we should adjust in the scenario. It is unknown in which direction these components could move. For example, it’s not clear whether sexual activity could increase or decrease, nor whether we could expect women would want to space/limit childbearing due to COVID-19, across all regions. There is an evidence for a postponement of childbearing in some countries, such as the study mentioned by reviewers for United States (Lindberg et al, 2020). The Aassve et al. (2020) paper hypothesised that fertility could move in different directions, depending on the development level of the country or within country.

Implicitly, our scenario assumes that the declines in users shift to the group of women with unmet need (since we assume no change in total demand). However, this does not account for the fact that some women who previously had no need for family planning (such as those who were planning a pregnancy) may shift into the group of women with unmet need. As we wanted to isolate only the potential impact of change in specific-method use on the demand for family planning satisfied by modern methods, we do not adjust possible changes in unmet need. We added this text in the Conclusions to strengthen this point:

*However, Aassve et al. (2020) hypothesized that - depending on the development level of the country – the changes in fertility preferences might not be present in all countries. Additionally, the frequency of sexual activity during COVID-19 pandemic could have been impacted by health and behavioural changes. We did not explicitly include the changes in fertility preferences and sexual activity in the scenario, since our main purpose was to illustrate the impact of method-specific access disruptions.*

**Method switching:**

We agree with reviewers that there could be some method-switching, and we added some text to the method section on this issue, since our scenario does not preclude the possibility of method-switching occurring. From the perspective of the SDG 3.7.1. indicator, method-switching between modern methods would not influence the indicator. For example, if all women who were not able to use injectables due to COVID-19 disruptions would use male condoms or pills instead, the overall number of modern users would remain unchanged. To implement the method-switching in the scenario, we would need to assume that the disruptions have also influenced the availability and accessibility of the methods to which women switch.  From a reproductive rights and choice perspective, women might not use methods that they wish to use. Additionally, method-switches are likely to be from more- to less-effective methods. This has crucial implications for prevention of unintended pregnancies and the implications for women who had to change to a less effective method because of the disruptions, rather than out of choice.

We added the following text in Methods:

*This does not mean there is no method-switching. Indeed, it is very possible that some women will switch from some methods to other methods, depending on their own context and the way that COVID-19 impacts specific methods in specific countries. Rather, the declines are an indication of the overall decline in use by method, after method-switching.*

**Specific country analysis and presentation of results:**

The analysis as presented was done at the regional and global level, and so we do not present country-specific scenarios, most especially because each country will be impacted differently and we do not wish to suggest that we know the disruptions across all countries included in the analysis. Instead, we have provided a framework with which countries themselves could run their own scenarios, adjusting the assumed declines in method-specific use according to the situations in each country. Estimates of use of contraceptive methods for all countries are available in the data booklet referenced in the paper (United Nations, 2019).

**The role of long-acting and permanent methods:**

We deleted the sentence “countries could improve access to long-acting methods because existing users of these methods are less impacted where there are disruptions to services”.

**Post-COVID-19 recovery:**

We added a comment in the conclusions around post-COVID-19 recovery and difference by methods.

*The COVID-19 pandemic has made the path towards achieving universal access to sexual and reproductive health-care services by 2030, including family planning, more uncertain. Once the disruptions due to COVID-19 are resolved, it is possible that contraceptive use – and therefore the SDG 3.7.1.  indicator – could return to the pre-disruption levels relatively quickly. For short-term method users, assuming that methods require frequent resupply, the health system could in principle recover to pre-COVID-19 levels within a short period of time, once service activities have fully resumed. However, for long-acting methods, there could be a longer period to catch up on services that were not provided during COVID-19 disruptions.*

------------------------------------

We have included information in the abstract on the range of declines assumed across methods, for the scenario presented in the paper.

We further elaborated the discussion of the potential impact of COVID-19 disruptions on the SDG 3.7.1. We also stressed the importance of the very real impact on the lives of women that even short-term disruptions in the access to contraception have.  Thank you for the suggestions on the wording.

*The COVID-19 pandemic has made the path towards achieving universal access to sexual and reproductive health-care services by 2030, including family planning, more uncertain. Once the disruptions due to COVID-19 are resolved, it is possible that contraceptive use – and therefore the SDG 3.7.1.  indicator – could return to the pre-disruption levels relatively quickly. […]*

*Most crucially, for women – and their partners and families – who experienced an unintended pregnancy resulting from the lack of access to contraception during COVID-19 disruptions, the impacts are long-lasting.*

# Peer review: Reviewer response for version 1

**DOI:** 10.21956/gatesopenres.14340.r29085

**Published:** 2020-07-30

**Authors:** Mengjia Liang

**Affiliations:** 1 Technical Division, United Nations Population Fund (UNFPA), New York City, NY, USA

This research note, published on Gates Open Research, presents the impact of COVID-19 pandemic on meeting contraceptive needs. The estimates are based on the 2020 Estimates and Projections of Family Planning Indicators published by the United Nations Population Division and assumptions of service disruption.

 I find the research note generally convincing and welcome, especially considering the pandemic is still rapidly evolving in many parts of the world. The paper is a considerable first step for more specific country analysis when country-specific service data become available, and has important policy implications.

 The scenario presented in the note assumes no change in sexual activity and total demand for contraceptives. This assumption may lead to an overestimate of the demand given previously sexually active women who are not married or living with a partner may experience a decrease in sexual activity, leading to lower or no demand for contraceptives during a lockdown. The research team may consider limiting the research scope to married or in-union women only, suggesting further assumptions for un-married/in union sexually active women, or providing a brief discussion of the implication. 

 The research team also makes no assumptions about the method switch, but no further details are provided. A brief discussion of the implication of this assumption would be desired.

 The authors indicate that a separate scenario using an assumed 10 percent decline in both short-term and long-acting reversible contraception is prepared as a sensitivity test. However, the results of this different scenario are not presented in the paper, nor are any comparisons and discussions provided.

 Lastly, in the conclusion section, the authors suggest that “countries could improve access to long-acting methods because existing uses of these methods are less impacted where are disruptions to services.” This statement may be premature as we don’t fully understand how the pandemic evolves in different parts of the world and how countries and local governments mitigate the risks. The suggestion may lead to unintended consequences, such as promoting long-acting methods even after the pandemic. Methods should be about women’s choices not to be limited by method availability.   

 Despite the limitation, the paper provides timely insights and reflects the global interest in women’s access to contraceptives in the pandemic. I would recommend the article for indexing with minor revisions.

Is the work clearly and accurately presented and does it cite the current literature?

Yes

If applicable, is the statistical analysis and its interpretation appropriate?

Not applicable

Are all the source data underlying the results available to ensure full reproducibility?

Yes

Is the study design appropriate and is the work technically sound?

Yes

Are the conclusions drawn adequately supported by the results?

Yes

Are sufficient details of methods and analysis provided to allow replication by others?

Yes

Reviewer Expertise:

Sexual and reproductive health and rights

I confirm that I have read this submission and believe that I have an appropriate level of expertise to confirm that it is of an acceptable scientific standard, however I have significant reservations, as outlined above.

# Response

**Published:** 2020-08-28

**Authors:** Vladimira Kantorova

**Affiliations:** United Nations, USA

Dear Mengjia, 

We thank you for the very helpful comments on the paper.

Because some of the issues were raised by more than one of the reviews, we reply to some of the common themes first. The remaining comments are included below.

**Changing total demand for family planning:**

All reviewers commented on the possibility of changing demand, either by changes in fertility preferences or sexual activity, or both. We agree with the reviewers that the changes in demand for family planning during COVID-19 could have substantial impact on the values of the indicator of demand for family planning satisfied by modern methods. We explored this at length, but decided this is not something we should adjust in the scenario. It is unknown in which direction these components could move. For example, it’s not clear whether sexual activity could increase or decrease, nor whether we could expect women would want to space/limit childbearing due to COVID-19, across all regions. There is an evidence for a postponement of childbearing in some countries, such as the study mentioned by reviewers for United States (Lindberg et al, 2020). The Aassve et al. (2020) paper hypothesised that fertility could move in different directions, depending on the development level of the country or within country.

Implicitly, our scenario assumes that the declines in users shift to the group of women with unmet need (since we assume no change in total demand). However, this does not account for the fact that some women who previously had no need for family planning (such as those who were planning a pregnancy) may shift into the group of women with unmet need. As we wanted to isolate only the potential impact of change in specific-method use on the demand for family planning satisfied by modern methods, we do not adjust possible changes in unmet need. We added this text in the Conclusions to strengthen this point:

*However, Aassve et al. (2020) hypothesized that - depending on the development level of the country – the changes in fertility preferences might not be present in all countries. Additionally, the frequency of sexual activity during COVID-19 pandemic could have been impacted by health and behavioural changes. We did not explicitly include the changes in fertility preferences and sexual activity in the scenario, since our main purpose was to illustrate the impact of method-specific access disruptions.*

**Method switching:**

We agree with reviewers that there could be some method-switching, and we added some text to the method section on this issue, since our scenario does not preclude the possibility of method-switching occurring. From the perspective of the SDG 3.7.1. indicator, method-switching between modern methods would not influence the indicator. For example, if all women who were not able to use injectables due to COVID-19 disruptions would use male condoms or pills instead, the overall number of modern users would remain unchanged. To implement the method-switching in the scenario, we would need to assume that the disruptions have also influenced the availability and accessibility of the methods to which women switch.  From a reproductive rights and choice perspective, women might not use methods that they wish to use. Additionally, method-switches are likely to be from more- to less-effective methods. This has crucial implications for prevention of unintended pregnancies and the implications for women who had to change to a less effective method because of the disruptions, rather than out of choice.

We added the following text in Methods:

*This does not mean there is no method-switching. Indeed, it is very possible that some women will switch from some methods to other methods, depending on their own context and the way that COVID-19 impacts specific methods in specific countries. Rather, the declines are an indication of the overall decline in use by method, after method-switching.*

**Specific country analysis and presentation of results:**

The analysis as presented was done at the regional and global level, and so we do not present country-specific scenarios, most especially because each country will be impacted differently and we do not wish to suggest that we know the disruptions across all countries included in the analysis. Instead, we have provided a framework with which countries themselves could run their own scenarios, adjusting the assumed declines in method-specific use according to the situations in each country. Estimates of use of contraceptive methods for all countries are available in the data booklet referenced in the paper (United Nations, 2019).

**The role of long-acting and permanent methods:**

We deleted the sentence “countries could improve access to long-acting methods because existing users of these methods are less impacted where there are disruptions to services”.

**Post-COVID-19 recovery:**

We added a comment in the conclusions around post-COVID-19 recovery and difference by methods.

*The COVID-19 pandemic has made the path towards achieving universal access to sexual and reproductive health-care services by 2030, including family planning, more uncertain. Once the disruptions due to COVID-19 are resolved, it is possible that contraceptive use – and therefore the SDG 3.7.1.  indicator – could return to the pre-disruption levels relatively quickly. For short-term method users, assuming that methods require frequent resupply, the health system could in principle recover to pre-COVID-19 levels within a short period of time, once service activities have fully resumed. However, for long-acting methods, there could be a longer period to catch up on services that were not provided during COVID-19 disruptions.*

-------------------------------------------

We agree with you that frequency of sexual activity during COVID-19 pandemic could have been impacted by health and behavioural changes. We also agree that in most settings, the frequency of sexual activity among unmarried women was potentially reduced more than among married women. Less frequent or no sexual intercourse would lead to lower demand for contraception.  Additionally, some women and their partners lacking access to modern contraception could abstain from sex because of the lack of access to safe and effective contraceptive methods. As with potential changes in demand for family planning, we did not consider it feasible to include the changes in sexual activity in our scenario illustrating the impact of method-specific disruptions. Hopefully, some of the surveys taking place during COVID-19 will ask the relevant question about sexual activity. will ask the question about sexual activity.

We added this text in Conclusions:

*However, Aassve et al. (2020) hypothesized that - depending on the development level of the country – the changes in fertility preferences might not be present in all countries. Additionally, the frequency of sexual activity during the COVID-19 pandemic could have been impacted by health and behavioural changes. We did not explicitly include the changes in fertility preferences and sexual activity in the scenario, since our main purpose was to illustrate the impact of method-specific access disruptions.*

The results of the scenario of 10% decline was shown in a footnote. We have now moved this to the main body of the paper – thanks for the suggestion.

# Peer review: Reviewer response for version 1

**DOI:** 10.21956/gatesopenres.14340.r29087

**Published:** 2020-07-28

**Authors:** Michelle Weinberger

**Affiliations:** 1 Avenir Health, Washington, DC, USA

This research note presents estimates of the potential impact of COVID-19 on global contraceptive use. It builds on estimates already published by others by (1) taking a global scope to include all countries of the world, and (2) quantifying the impact on SDG indicator 3.7.1 the proportion of women who have their need for family planning satisfied by modern methods.

 As acknowledged by the authors, they present results based on a simplified set of assumptions to provide illustrative results of the potential impact of COVID-19 on the achievement of SDG 3.7.1. These results provide useful evidence to support advocacy efforts for the importance of sustaining access to contraception during COVID-19. While there is value in such a simple set of assumptions/scenarios, there could also be benefit in the authors exploring some additional considerations, for example:

- Including a starker scenario in their sensitivity testing to explore a full range of possibilities given the large uncertainty about the potential impacts of COVID-19 (so that the detailed scenario presented in the paper is a middle ground between the 10% declines and some higher level of decline). This would be useful, especially as in the introduction the authors note that existing scenarios may provide “conservative estimates” of impact.
- Quantifying the impact of potential increases in demand as a result of COVID-19. The authors acknowledge fertility preferences may change in response to COVID-19. While there is not yet data to understand these changes, such changes would lead to changes in the denominator of the demand satisfied indicator. The paper already explores changes to the numerator (mCP). Adding a simple set of assumptions to quantify potential denominator changes would provide useful context, especially as a focus on demand satisfied is a unique aspect of this research note. 

Finally, a few more specific notes regarding the research note:

- It may help to add some detail to the methods section to more closely follow the calculations shown in the Excel file. For example, that method mix is is applied to the projected CP in 2020 to estimate method prevalence, then prevalence by method is reduced based on the %s in Table 1, then a new mCP is constructed and divided by the 2020 total demand projection to estimate a new demand satisfied measure. 
- In discussing the declines in demand satisfied in the results the numbers presented are percentage point declines, not percent changes. The language should be edited to clarify this. 
- The authors may wish to cite existing guidance (from groups such as WHO, FIGO and FSRH CEU) in the final paragraph of their conclusion on the use of self-care methods and other strategies to minimize the impact of COVID-19.   
- Given the focus on an SDG indicator, the authors may wish to add some text to the conclusion about how COVID-19 may impact on 2030 SDG achievement; do they hypothesize these changes to be short term, or, potentially have lasting impacts on trajectories towards 2030? 

Is the work clearly and accurately presented and does it cite the current literature?

Yes

If applicable, is the statistical analysis and its interpretation appropriate?

Yes

Are all the source data underlying the results available to ensure full reproducibility?

Yes

Is the study design appropriate and is the work technically sound?

Yes

Are the conclusions drawn adequately supported by the results?

Partly

Are sufficient details of methods and analysis provided to allow replication by others?

Partly

Reviewer Expertise:

family planning, modeling health impact

I confirm that I have read this submission and believe that I have an appropriate level of expertise to confirm that it is of an acceptable scientific standard, however I have significant reservations, as outlined above.

# Response

**Published:** 2020-08-28

**Authors:** Vladimira Kantorova

**Affiliations:** United Nations, USA

Dear Michelle,

We thank you for the very helpful comments on the paper.

Because some of the issues were raised by all three reviews, we reply to some of the common themes first. The remaining comments are included below.

**Changing total demand for family planning:**

All reviewers commented on the possibility of changing demand, either by changes in fertility preferences or sexual activity, or both. We agree with the reviewers that the changes in demand for family planning during COVID-19 could have substantial impact on the values of the indicator of demand for family planning satisfied by modern methods. We explored this at length, but decided this is not something we should adjust in the scenario. It is unknown in which direction these components could move. For example, it’s not clear whether sexual activity could increase or decrease, nor whether we could expect women would want to space/limit childbearing due to COVID-19, across all regions. There is an evidence for a postponement of childbearing in some countries, such as the study mentioned by reviewers for United States (Lindberg et al, 2020). The Aassve et al. (2020) paper hypothesised that fertility could move in different directions, depending on the development level of the country or within country.

Implicitly, our scenario assumes that the declines in users shift to the group of women with unmet need (since we assume no change in total demand). However, this does not account for the fact that some women who previously had no need for family planning (such as those who were planning a pregnancy) may shift into the group of women with unmet need. As we wanted to isolate only the potential impact of change in specific-method use on the demand for family planning satisfied by modern methods, we do not adjust possible changes in unmet need. We added this text in the Conclusions to strengthen this point:

*However, Aassve et al. (2020) hypothesized that - depending on the development level of the country – the changes in fertility preferences might not be present in all countries. Additionally, the frequency of sexual activity during COVID-19 pandemic could have been impacted by health and behavioural changes. We did not explicitly include the changes in fertility preferences and sexual activity in the scenario, since our main purpose was to illustrate the impact of method-specific access disruptions.*

**Method switching:**

We agree with reviewers that there could be some method-switching, and we added some text to the method section on this issue, since our scenario does not preclude the possibility of method-switching occurring. From the perspective of the SDG 3.7.1. indicator, method-switching between modern methods would not influence the indicator. For example, if all women who were not able to use injectables due to COVID-19 disruptions would use male condoms or pills instead, the overall number of modern users would remain unchanged. To implement the method-switching in the scenario, we would need to assume that the disruptions have also influenced the availability and accessibility of the methods to which women switch.  From a reproductive rights and choice perspective, women might not use methods that they wish to use. Additionally, method-switches are likely to be from more- to less-effective methods. This has crucial implications for prevention of unintended pregnancies and the implications for women who had to change to a less effective method because of the disruptions, rather than out of choice.

We added the following text in Methods:

*This does not mean there is no method-switching. Indeed, it is very possible that some women will switch from some methods to other methods, depending on their own context and the way that COVID-19 impacts specific methods in specific countries. Rather, the declines are an indication of the overall decline in use by method, after method-switching.*

**Specific country analysis and presentation of results:**

The analysis as presented was done at the regional and global level, and so we do not present country-specific scenarios, most especially because each country will be impacted differently and we do not wish to suggest that we know the disruptions across all countries included in the analysis. Instead, we have provided a framework with which countries themselves could run their own scenarios, adjusting the assumed declines in method-specific use according to the situations in each country. Estimates of use of contraceptive methods for all countries are available in the data booklet referenced in the paper (United Nations, 2019).

**The role of long-acting and permanent methods:**

We deleted the sentence “countries could improve access to long-acting methods because existing users of these methods are less impacted where there are disruptions to services”.

**Post-COVID-19 recovery:**

We added a comment in the conclusions around post-COVID-19 recovery and difference by methods.

*The COVID-19 pandemic has made the path towards achieving universal access to sexual and reproductive health-care services by 2030, including family planning, more uncertain. Once the disruptions due to COVID-19 are resolved, it is possible that contraceptive use – and therefore the SDG 3.7.1.  indicator – could return to the pre-disruption levels relatively quickly. For short-term method users, assuming that methods require frequent resupply, the health system could in principle recover to pre-COVID-19 levels within a short period of time, once service activities have fully resumed. However, for long-acting methods, there could be a longer period to catch up on services that were not provided during COVID-19 disruptions.*

------------------------------------------

For comparison, we have prepared a starker “high disruption” scenario, by using the assumptions of “high” percent reduction in service utilization from UNFPA (2020), using public sector high decline (e.g. 40% decline for methods requiring service provision, 20% decline for methods that can be sourced from alternative places). Under the “high disruption” scenario, the estimate of the need for family planning satisfied with modern methods in 2020 could fall to 65%. In sub-Saharan Africa, the estimate for year 2020 would be 42%, as compared to 55% in pre-COVID situation.

All scenarios are purely illustrative and the “high disruption” scenario gives information about an upper range of possible impact. While for some regions, the “high disruption” scenario is indeed within the range of possibilities, in other regions the impact could be smaller. Therefore, we suggest not to present a global value in the paper and not to include the regional estimates in the figure. Such calculations can be easily done in *Underlying data.*

We have added more detail to the methods section to more closely follow the calculations shown in the Excel file, thank you for the suggestion:

*We applied the method-mix (United Nations 2019) to the contraceptive prevalence in 2020 (United Nations, 2020a), to produce estimates of method-specific prevalence for 2020. These were then reduced by method-specific declines (Table 1). The resulting modern contraceptive prevalence was divided by total demand (United Nations, 2020a), in order to produce a scenario of demand for family planning satisfied by modern methods.*

We clarified in the results section the numbers presented are percentage point declines. Thank you for pointing that out.

We added reference in the conclusions to the existing guidance from WHO, in particular, *Maintaining essential health services: operational guidance for the COVID-19 context *recommending practical actions that countries can take at national, subregional and local levels.

The text now reads:

*WHO recommends practical actions that countries can take at national, subregional and local levels to reorganize and safely maintain access to high-quality, essential health services in the pandemic context (WHO, 2020; on page 29), such as relaxation of requirements for a prescription for oral or self-injectable contraception and emergency contraception and provision of multimonth supplies with clear information about the method and how to access referral care for adverse reactions;, and enabling pharmacies and drugstores to increase the range of contraceptive options they can provide. For the transition towards restoration of the services, it recommends to plan for clients whose choice are longer-term methods (such as IUDs, implants) or permanent methods, if these services were disrupted.*
